# Supplementary material for: CD13 orients the apical-basal polarity axis necessary for lumen formation
Source: Nat Commun. 2021 Aug 4;12:4697. doi: 10.1038/s41467-021-24993-x (PMC8338993; doi:10.1038/s41467-021-24993-x)

CD13 orients the apical-basal polarity axis necessary for lumen formation

Li-Ting Wang, Abira Rajah, Claire M Brown, Luke McCaffrey*

SUPPLEMENTARY INFORMATION

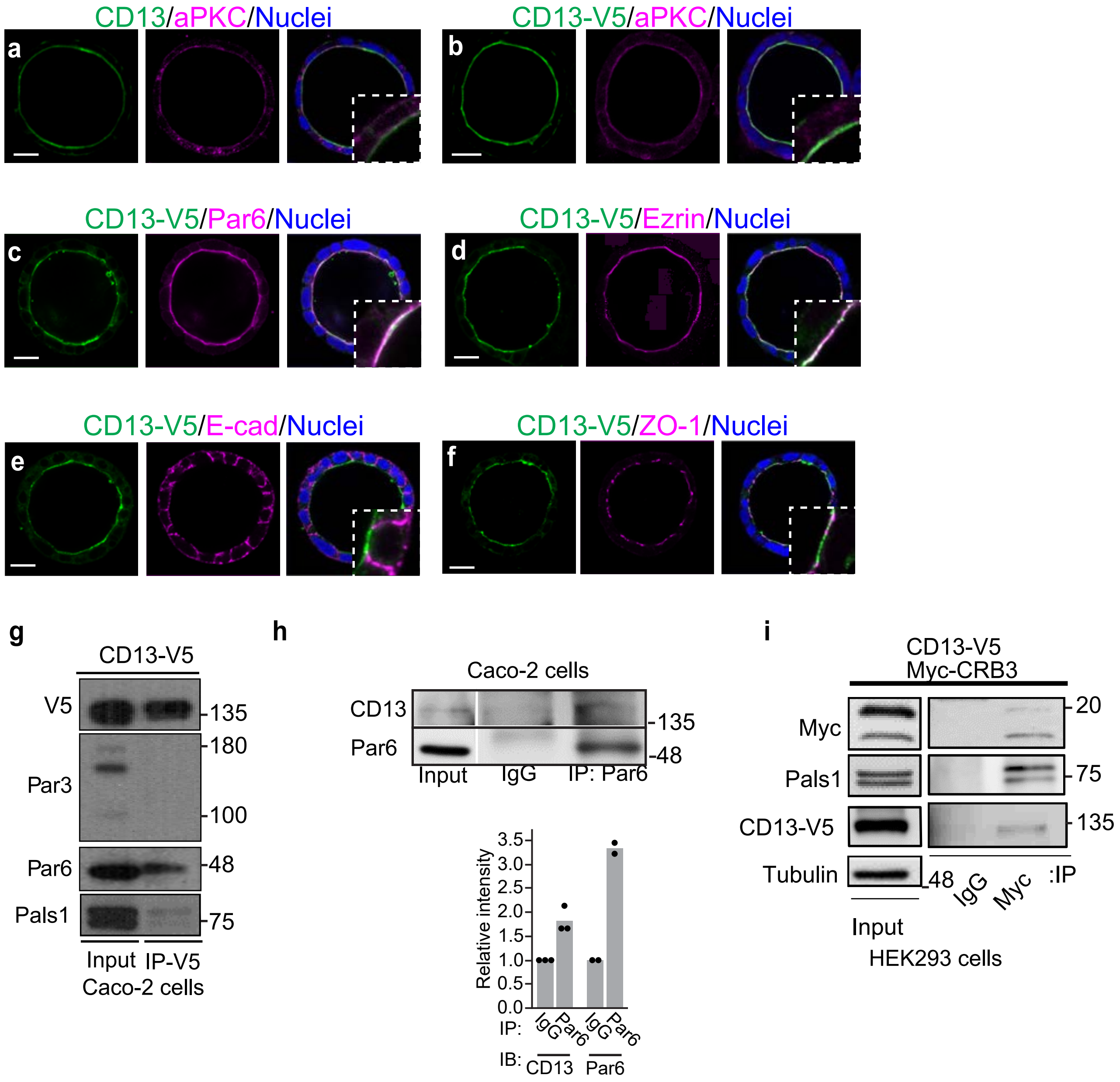

Supplemental Figure 1. **CD13 associates with apical proteins and localizes at the apical domain of 3D Caco-2 cysts. Associated with Figure 1. a** Confocal images of polarized 3D Caco-2 cysts cultured for 10 days then immunostained for endogenous CD13 (green) and apical aPKC (magenta). **b-f** Representative confocal images of polarized 3D Caco-2 cysts immunostained for V5-tagged CD13 (green) and polarity markers (magenta) aPKC (b), Par6 (**c**), Ezrin (**d**), E-cad (**e**), and ZO-1 (**f**). **g** Immunoprecipitation was performed with anti-V5 in stably expressed CD13-V5 Caco-2 cells and blotted for V5, Par3, Par6, and Pals1. **h** Immunoprecipitation was performed in Caco-2 lysates with IgG or anti-Par6 antibody and blotted for endogenous CD13 and Par6. The lower graph shows quantification of band intensities from three independent experiments. **i** Co-immunoprecipitation of CD13 and CRB3 was performed with anti-IgG or anti-myc in HEK293 cells. The presence of CD13 in immunoprecipitates was determined by western blot analysis using anti-V5. Images are representative from three independent experiments. Bars: **a-f**, 20µm.

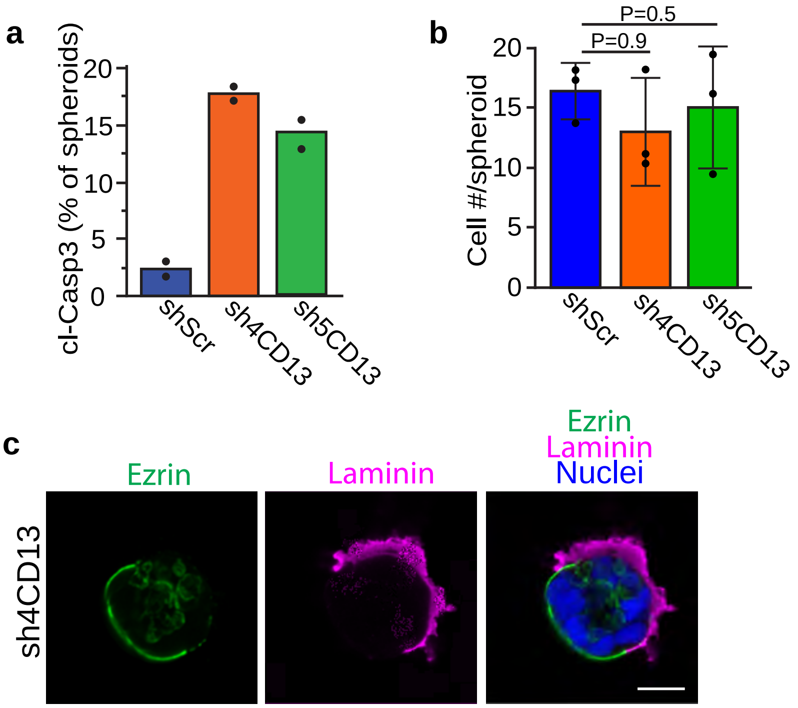

Supplemental Figure 2. **CD13 knockdown disrupts the orientation of apical-basal polarity. Associated with Figure 2. a** Caco-2 were cultured for 10 days and the proportion of spheroids with one or more apoptotic cells (measured by cleaved Caspase) were quantified from n=7 fields of view for each condition. Individual dots represent mean values from independent experiments. **b** Quantification of the mean number of cells per spheroid +/- SEM for the indicated conditions (n=201 shScr, n=242 sh4CD13, n=208 sh5CD13 spheroids) after 10 days in culture. Dots represent mean values from independent experiments. The p-values were determined by unpaired two-tailed student's t-test. **c** Confocal images of 3D Caco-2 spheroids immunostained for Ezrin (green) and laminin (magenta). Images are representative from least three independent experiments. Bars: **c**, 30μm.

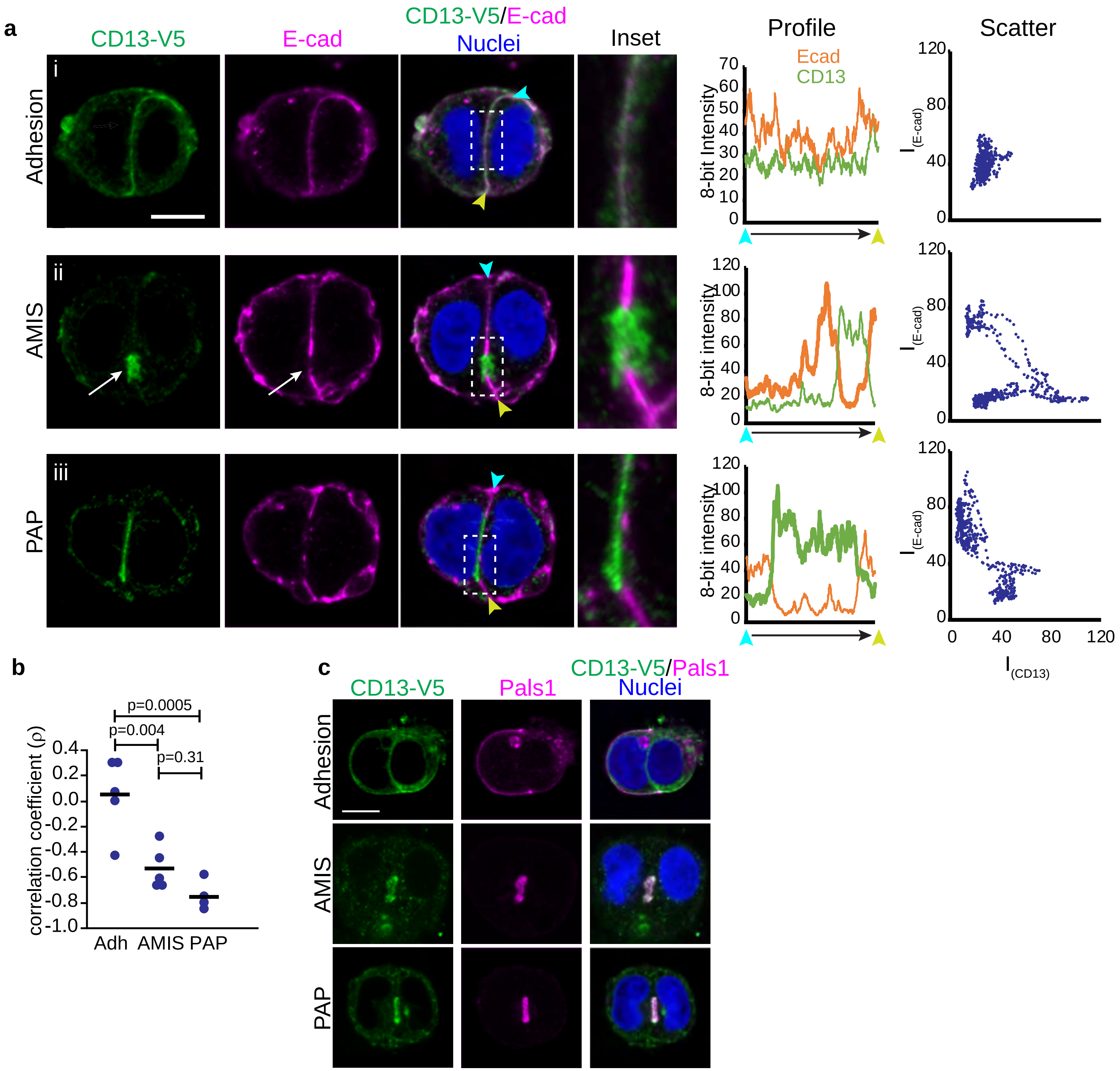

Supplemental Figure 3. **Recruitment of CD13 to focal sites is coincident with E-cadherin displacement during polarization in Caco-2 cells. Associated with Figure 3. a** Confocal images for CD13-V5 (green) and E-cad (magenta) showing the relative localization of CD13 and E-cad at adhesion, AMIS, and PAP stage in two cell structures of Caco-2 cysts. Graphic profile depicting changes in fluorescent intensity (8-bit intensity range: 0-255) from blue to yellow arrows. Adjacent scatter plots showing the relationships between CD13 and E-cad. **b** Quantification of the correlation coefficient of CD13 and E-cad at adhesion, AMIS, and PAP stage in two cell structures of 3D Caco-2 cysts. p-values were determined by unpaired two-tailed student's t-test. **c** Confocal images of 2-cell Caco-2 structures immunostained for CD13-V5 (green) and Pals1 (magenta) showing their localization during polarity initiation. Images are representative from three independent experiments (**a,c**). Bars: 10μm.

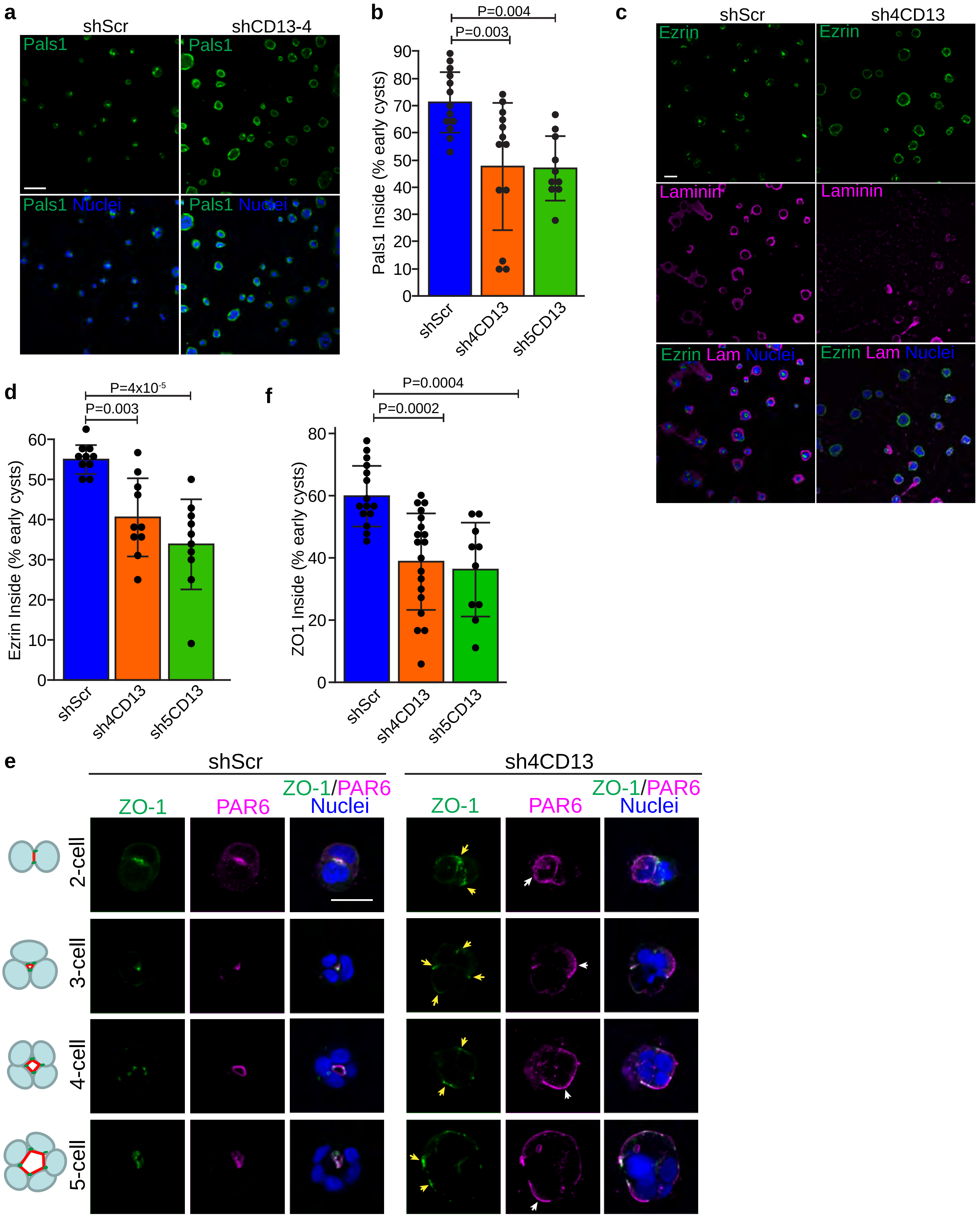

Supplementary Figure 4. Figure legend on following page.

Supplemental Figure 4. **CD13 depletion disrupts early polarization in 3D Caco-2 cysts. Associated with Figure 4. a** Low-power confocal images of control (shScr) and CD13 knock-down (sh4CD13) 3D Caco-2 cells cultured for 2 days then immunostained for Pals1 (green). **b** Quantification of the percentage of Caco2 structures with Palsl localize to the inside of shScr (n=235), sh4CD13 knock-down (n=326) and sh5CD13 knock-down (n=154) in 3D Caco-2 cysts at early stages. Dots represent means from independent experiments. **c** Confocal images of control (shScr) and CD13 knock-down (sh4CD13) 3D Caco-2 cells cultured for 2 days then immunostained for Ezrin (green) and laminin (magenta). **d** Quantification of the percentage Caco-2 structures with Ezrin localization to the inside for shScr (n=153), sh4-CD13 knock-down (n=162) and sh5-CD13 knock-down (n=147) cells. Dots represent means from independent experiments. **e** Confocal images of control (shScr) and CD13 knock-down (sh4CD13) 3D Caco-2 cysts cultured for 2 days then immunostained for tight junction marker, ZO1 (green) and Par6 (magenta). **f** Quantification of the percentage of Caco-2 structures with ZO-1 localized to the inside of shScr (n=302), sh4CD13 knock-down (n=278) and sh5CD13 knock-down (n=146) cells. Dots represent means from independent experiments. p-values were determined by unpaired two-tailed student's t-test (**b,d,f**). Images are representative from three independent experiments. Bars: **a,c**, 100μm; e, 30μm.

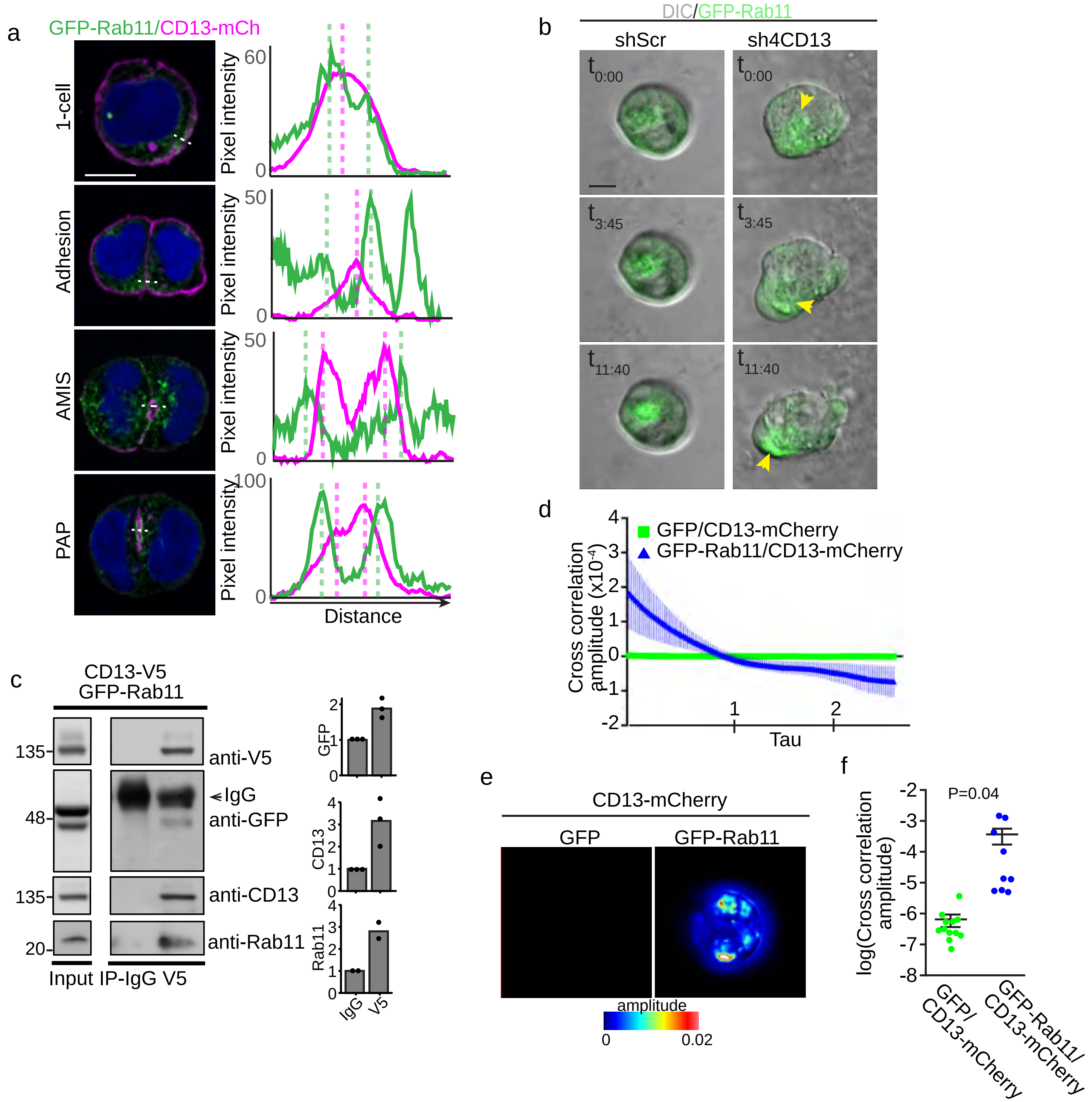

Supplementary Figure 5. **Knockdown CD13 disrupts Rab11-dependent early polarization in Caco-2 cells. Associated with Figure 5. a** Confocal images showing the localization of CD13-mCherry (magenta) and GFP-Rab11 (green) at 1-cell, and adhesion, AMIS, and PAP stage in 2-cell Caco-2 structures from Fig. 5A with local intensity profiles of CD13 and Rab11 (right panels). Vertical lines show adjoining but non-overlapping CD13 and Rab11 intensity peaks. **b** DIC/confocal images showing selected frames from time-lapse series of shScr and sh4CD13 3D Caco-2 structures. Images were captured every 25 min. **c** Co-immunoprecipitation of CD13-V5 and GFP-Rab11 was performed with IgG or anti-V5 in HEK293 cells. The presence of Rab11 in immunoprecipitates was determined by western blot analysis using anti-GFP and anti-Rab11. Graphs on the right show quantification of band intensities from two independent experiments. **d** Relative cross correlation amplitude profile of cells co-expressing GFP or GFP-Rab11 and CD13-mCherry fusions over time. Tau represents the time separation between two acquired images. **e** Co-binding intensity profile of Caco-2 cells co-expressing GFP (n=12) or GFP-Rab11 (n=9) with CD13-mCherry. **f** Relative cross correlation amplitude profile of cells co-expressing GFP (n=18) or GFP-Rab11 (n=17) with CD13-mCherry fusions at the first time-point. Data are presented as mean values +/- SEM. p-values were determined by unpaired two-tailed student's t-test. Images are representative from three independent experiments. Bars: 10μm.

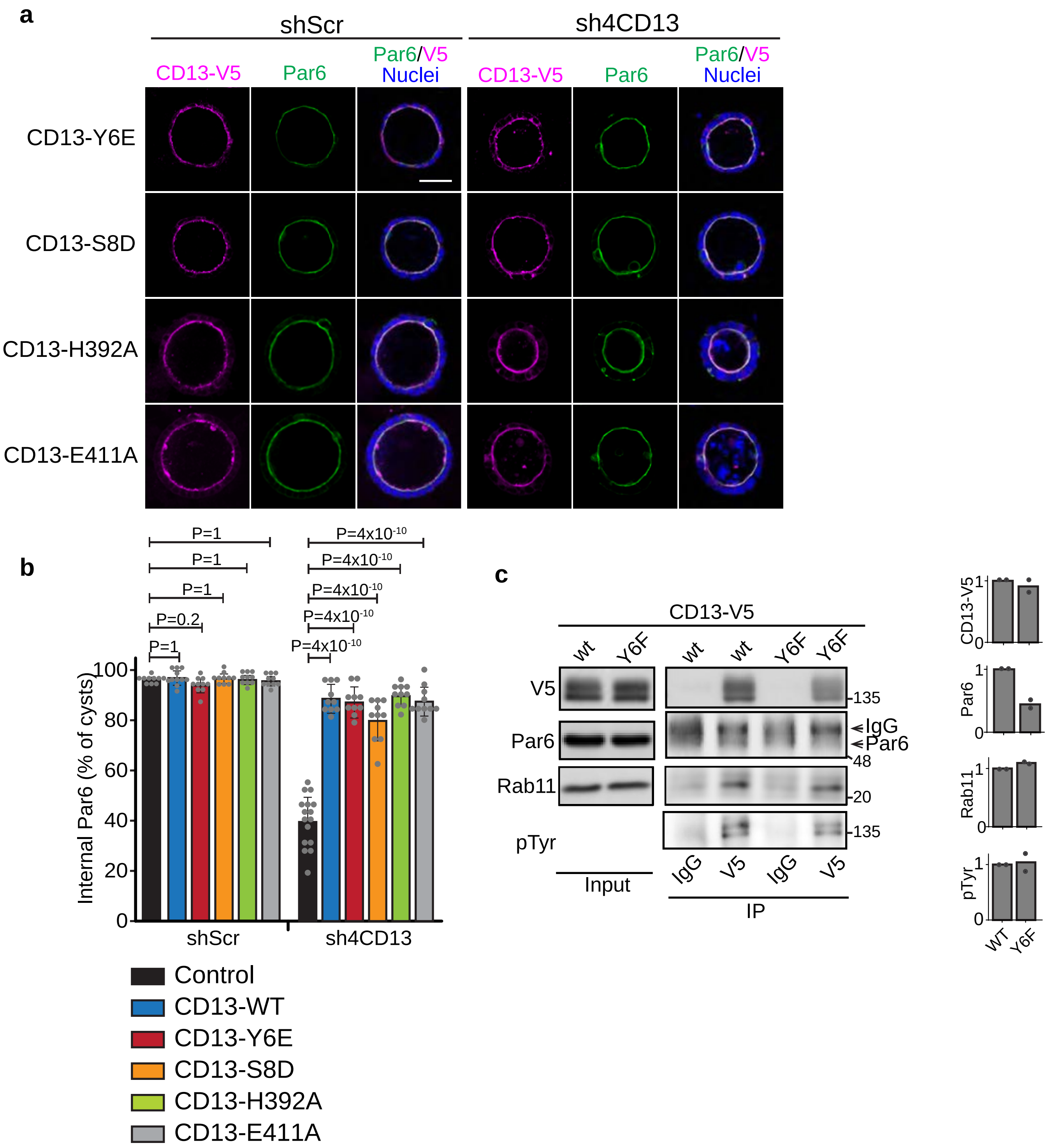

Supplemental Figure 6. **The intracellular domain of CD13 is required to establish apical-basal polarity. Associated with Figure 7. a** Confocal images of Caco-2 cells with control (shScr) or CD13 knockdown (sh4CD13) cells expressing CD13 mutants immunostained for CD13-V5 (magenta) and Par6 (green). Images are representative from two independent experiments. **b** Quantification of the percentage of Par6 internally in wildtype (shScr, n=515; shCD13, n=292 cell structures), CD13-Y6E (shScr, n=555; shCD13, n=285 cell structures), CD13-S8D (shScr n=400; shCD13, n=337 cell structures), CD13-H392A (shScr, n=475; shCD13, n=298 cell structures), and CD13-E411A (shScr, n=513; shCD13, n=283 cell structures) for shScr and shCD13 Caco-2 cysts. Data are presented as mean values +/- SD. Comparisons of multiple means was performed by ANOVA using Tukey's post-hoc test. **c** Immunoprecipitation was performed with anti-IgG or anti-V5 in stably expressed CD13-wild type or CD13-Y6F Caco-2 cells. Immunoprecipitates were blotted for V5, Par6, Rab11 and pan-phosphotyrosine. Graphs to the right show quantification of band intensity from two independent experiments. Bars: **a**, 50μm.

Supplementary Table 1. **shRNA sequences.**

shRNA Name	Sequence
sh4CD13	CCGGCCACAGCAAGAAGCTCAACTACTCGAGTAGTTGAGCTTCTTGCTGTGGTTTTTG
sh5CD13	CCGGGTGACCATAGAGTGGTGGAATCTCGAGATTCCACCACTCTATGGTCACTTTTTG
sh1Rab11A	CCGGATCATGCTGATAGTAACATTGCTCGAGCAATGTTACTATCAGCATGATTTTTTG
sh4Rab11A	GTACCGGTATCATGCTTGTGGGCAATAACTCGAGTTATTGCCCACAAGCATGATATTTTTTG
sh1Rab35	CCGGAGACGGAAGATGCCTACAAATCTCGAGATTTGTAGGCATCTTCCGTCTTTTTTG
sh5Rab35	GTACCGGTTCACGAAATCAACCAGAACTCTCGAGAGTTCTGGTTGATTTCGTGAATTTTTTG

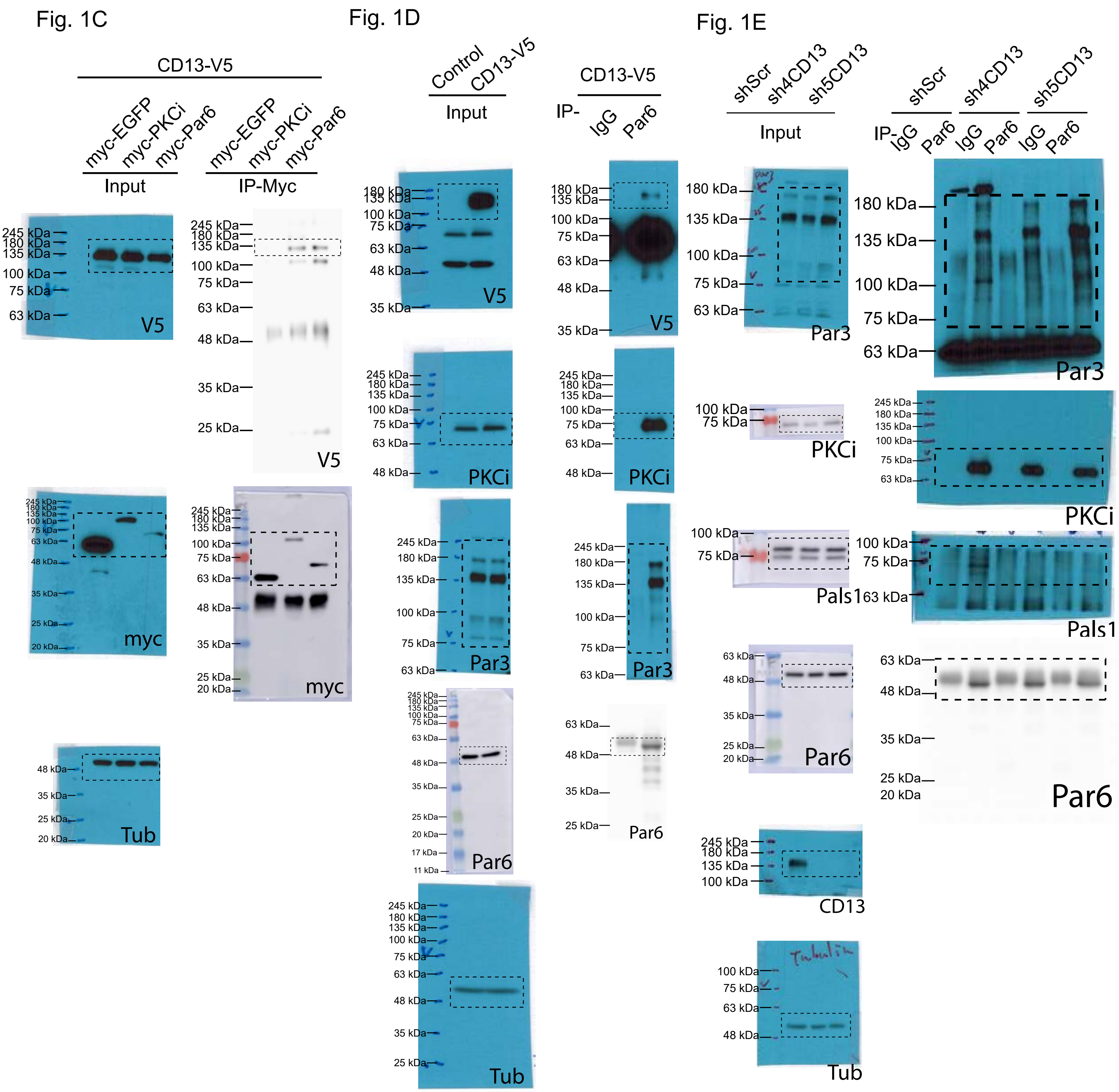

Fig. 1C
CD13-V5
myc-EGFP
myc-PKCi
myc-Par6
Input
IP-Myc
V5
myc
Tub
Fig. 1D
Control
CD13-V5
Input
IP-
IgG
Par6
V5
PKCi
Par3
Par6
Tub
Fig. 1E
shScr
sh4CD13
sh5CD13
Input
IP-
IgG
Par6
Par3
PKCi
Pals1
Par6
CD13
Tub
245 kDa
180 kDa
135 kDa
100 kDa
75 kDa
63 kDa
48 kDa
35 kDa
25 kDa
20 kDa
17 kDa
11 kDa

Fig. 1G

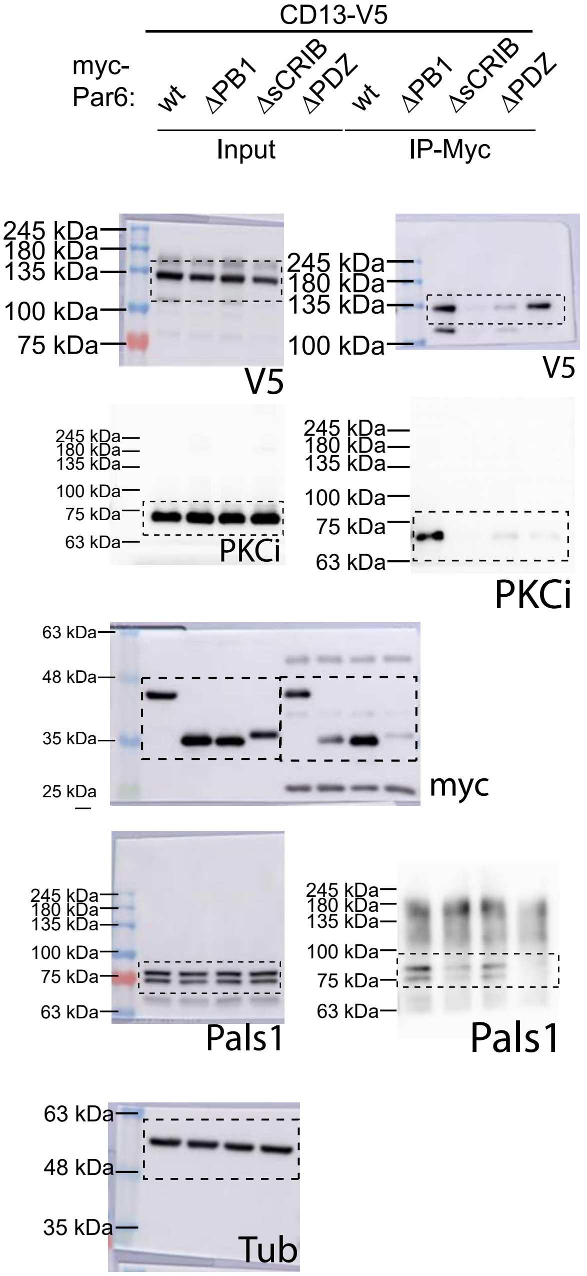
CD13-V5
myc-Par6:
wt
ΔPB1
ΔsCRIB
ΔPDZ
wt
ΔPB1
ΔsCRIB
ΔPDZ
Input
IP-Myc
245 kDa
180 kDa
135 kDa
100 kDa
75 kDa
V5
V5
PKCi
PKCi
63 kDa
48 kDa
35 kDa
25 kDa
myc
Pals1
Pals1
Tub

Fig. 1H

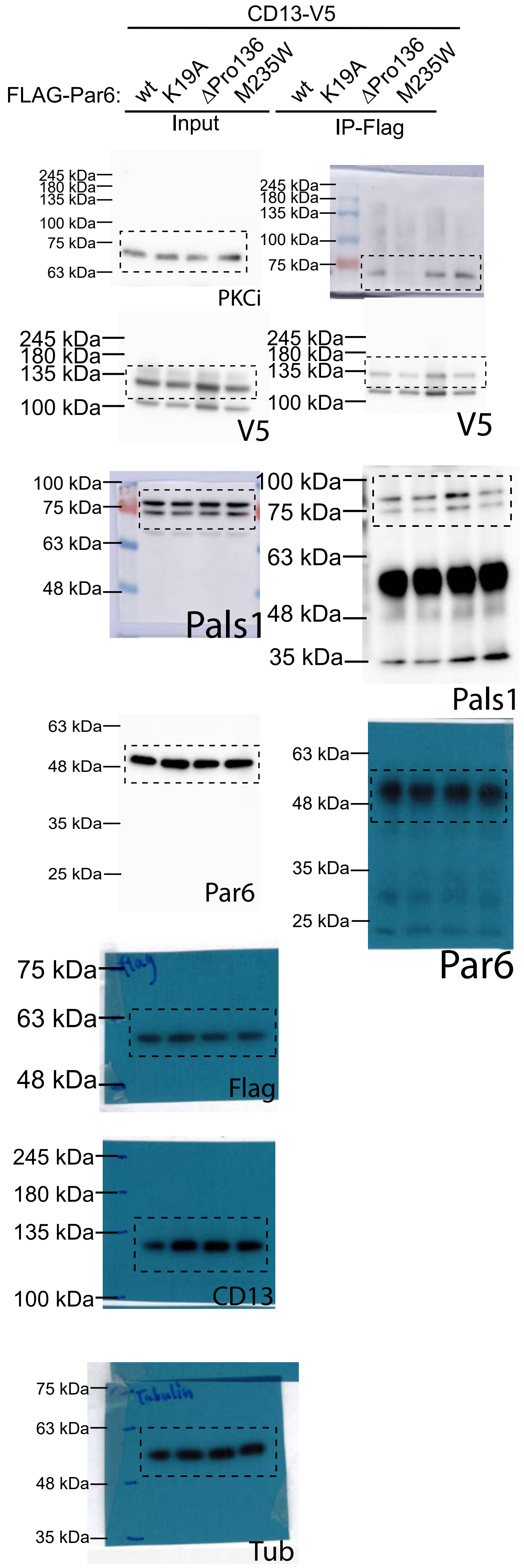
CD13-V5
FLAG-Par6:
wt
K19A
ΔPro136
M235W
wt
K19A
ΔPro136
M235W
Input
IP-Flag
245 kDa
180 kDa
135 kDa
100 kDa
75 kDa
63 kDa
PKCi
V5
V5
48 kDa
35 kDa
25 kDa
Pals1
Pals1
Par6
Par6
Flag
CD13
Tub

Supplementary Figure S1

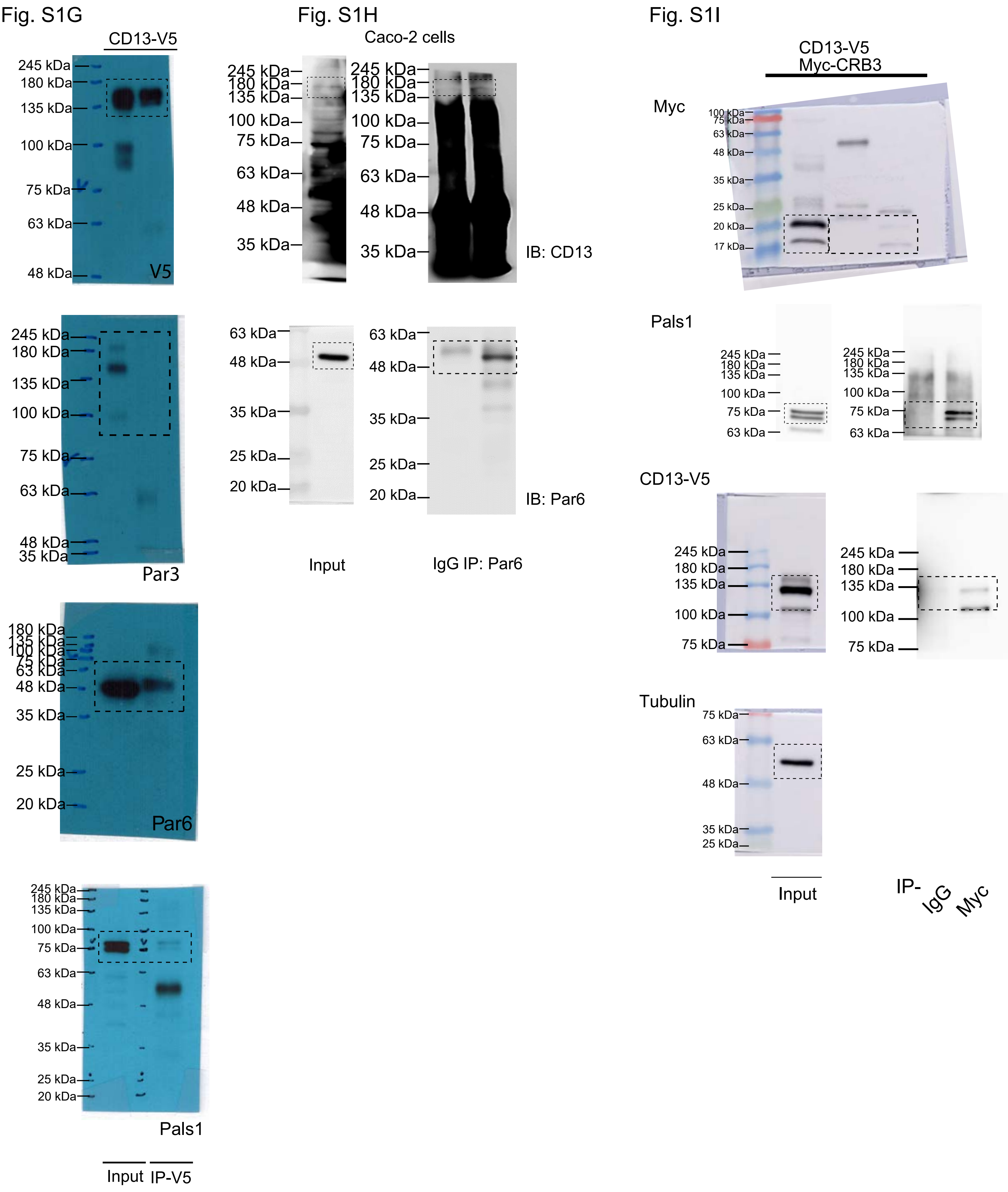

Supplementary Figure S5

Fig. S5C

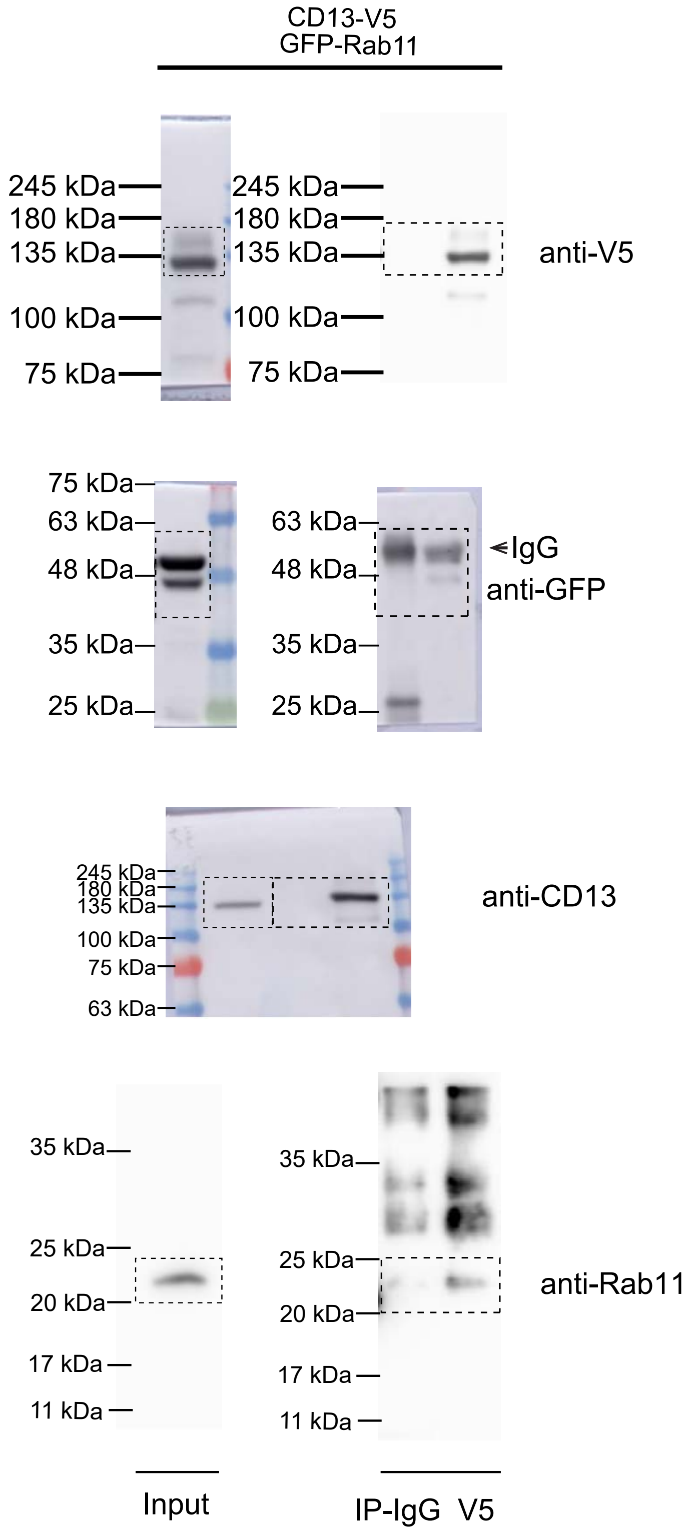

Supplementary Figure S6

Fig. S6C

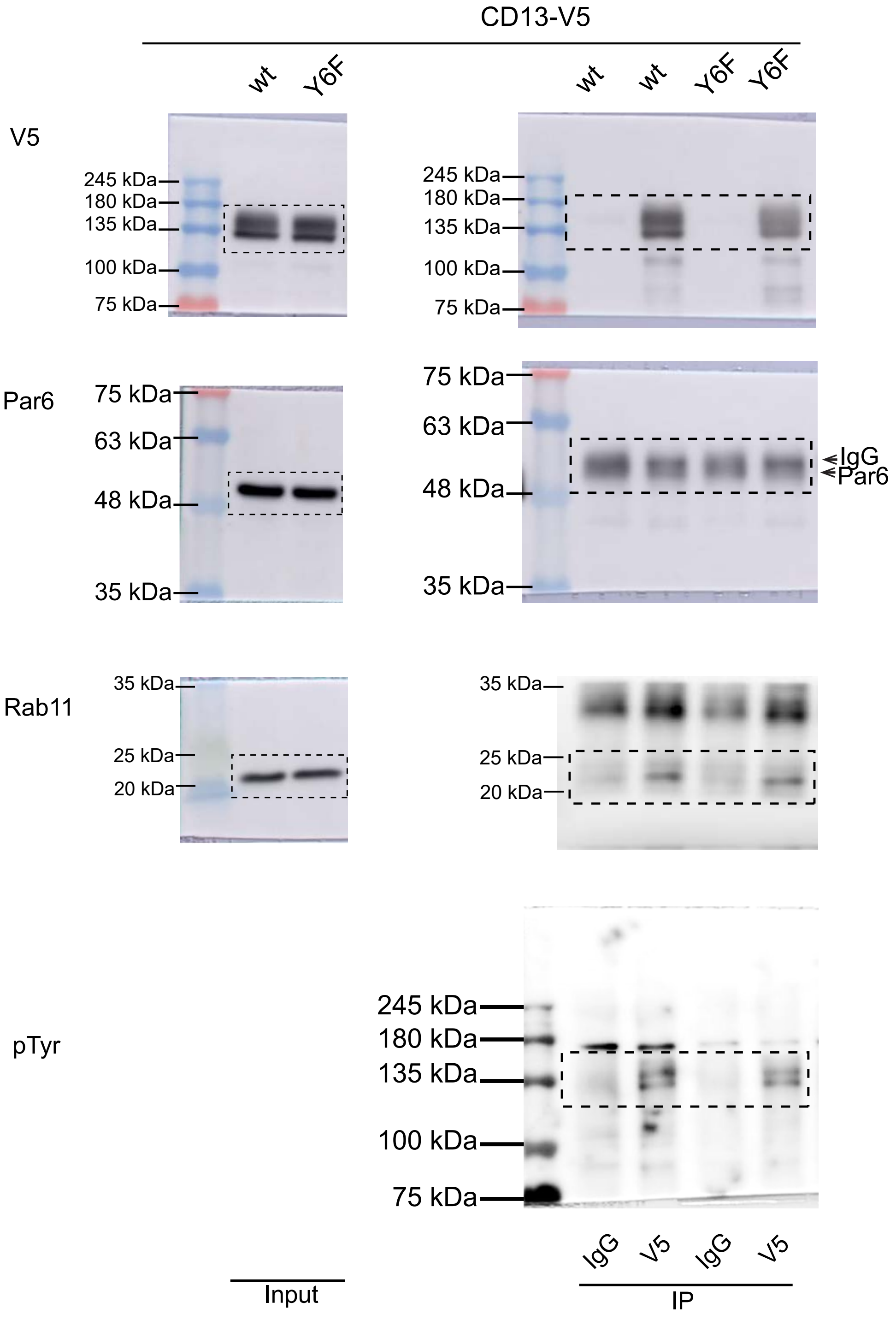

Supplement: Supplementary file 1 — Supplementary Information [file 41467_2021_24993_MOESM1_ESM.pdf]
