# Supplementary material for: CD13 orients the apical-basal polarity axis necessary for lumen formation
Source: Nat Commun. 2021 Aug 4;12:4697. doi: 10.1038/s41467-021-24993-x (PMC8338993; doi:10.1038/s41467-021-24993-x)
Supplement: Supplementary file 3 — Description of Additional Supplementary Files [file 41467_2021_24993_MOESM3_ESM.pdf]

Description of Additional Supplementary Files

File Name: Supplementary Movie 1
Description: Time-lapse series of Caco-2 cell polarization and lumen formation showing CD13-mCherry (magenta) and GFP-Par6 (green).

File Name: Supplementary Movie 2
Description: Time-lapse series of control (shScr) Caco-2 cells showing GFPRab11 (green) and phase-contrast during apical membrane initiation.

File Name: Supplementary Movie 3
Description: Confocal time-lapse series of CD13-depleted (shCD13-4) Caco-2 cells showing GFP-Rab11 (green) and phase- contrast during apical membrane initiation.
