# Supplementary material for: CD13 orients the apical-basal polarity axis necessary for lumen formation
Source: Nat Commun. 2021 Aug 4;12:4697. doi: 10.1038/s41467-021-24993-x (PMC8338993; doi:10.1038/s41467-021-24993-x)
Supplement: Supplementary file 7 — Reporting Summary [file 41467_2021_24993_MOESM7_ESM.pdf]

nature portfolio

Corresponding author(s): Luke McCaffrey
Last updated by author(s): Jun 24, 2021

Reporting Summary

Nature Portfolio wishes to improve the reproducibility of the work that we publish. This form provides structure for consistency and transparency in reporting. For further information on Nature Portfolio policies, see our Editorial Policies and the Editorial Policy Checklist.

Statistics

For all statistical analyses, confirm that the following items are present in the figure legend, table legend, main text, or Methods section.

n/a	Confirmed	
☐	☒	The exact sample size (n) for each experimental group/condition, given as a discrete number and unit of measurement
☐	☒	A statement on whether measurements were taken from distinct samples or whether the same sample was measured repeatedly
☐	☒	The statistical test(s) used AND whether they are one- or two-sided<br>*Only common tests should be described solely by name; describe more complex techniques in the Methods section.*
☒	☐	A description of all covariates tested
☐	☒	A description of any assumptions or corrections, such as tests of normality and adjustment for multiple comparisons
☐	☒	A full description of the statistical parameters including central tendency (e.g. means) or other basic estimates (e.g. regression coefficient) AND variation (e.g. standard deviation) or associated estimates of uncertainty (e.g. confidence intervals)
☐	☒	For null hypothesis testing, the test statistic (e.g. F, t, r) with confidence intervals, effect sizes, degrees of freedom and P value noted<br>*Give P values as exact values whenever suitable.*
☒	☐	For Bayesian analysis, information on the choice of priors and Markov chain Monte Carlo settings
☒	☐	For hierarchical and complex designs, identification of the appropriate level for tests and full reporting of outcomes
☐	☒	Estimates of effect sizes (e.g. Cohen's d, Pearson's r), indicating how they were calculated

Our web collection on statistics for biologists contains articles on many of the points above.

Software and code

Policy information about availability of computer code

Data collection	Microscopy images were captured using ZEN 2012 version 14.0.0.0 software. TIRF data were captured using Meta Morph 7.1 image acquisition software (Molecular Devices Inc.).
Data analysis	The statistical analyses were performed with Microsoft Excel office 365, Graphpad Prism 6, JMP 14.3., and MATLAB R2020b. Images analysis was performed using FIJI/Image J vl.53e. TIRF data were performed using SimFCS 4 software (Globals Software, Laboratory for Fluorescence Dynamics, Irvine, CA).

For manuscripts utilizing custom algorithms or software that are central to the research but not yet described in published literature, software must be made available to editors and reviewers. We strongly encourage code deposition in a community repository (e.g. GitHub). See the Nature Portfolio guidelines for submitting code & software for further information.

Data

Policy information about availability of data

All manuscripts must include a data availability statement. This statement should provide the following information, where applicable:

- Accession codes, unique identifiers, or web links for publicly available datasets
- A description of any restrictions on data availability
- For clinical datasets or third party data, please ensure that the statement adheres to our policy

Source data are provided with this paper. The image datasets generated and analyzed during the current study are available from the corresponding author on request.

Field-specific reporting

Please select the one below that is the best fit for your research. If you are not sure, read the appropriate sections before making your selection.

- [x] Life sciences
- [ ] Behavioural & social sciences
- [ ] Ecological, evolutionary & environmental sciences

For a reference copy of the document with all sections, see nature.com/documents/nr-reporting-summary-flat.pdf

Life sciences study design

All studies must disclose on these points even when the disclosure is negative.

Sample size	Sample size was determined based on previous studies by our group and are similar to those used by other groups using the same cell line (PMIDs 32665580, 33850145). The sample sizes used were adequate to obtain clear results based on the magnitude and consistency of measurable differences between experimental groups.
Data exclusions	No data were excluded.
Replication	We performed at least two to three independent experiments with multiple cell structures analyzed for each. All attempts at replication were successful. The number of cell structures analyzed and number of independent experiments are indicated in the figure legends and available in the source data file.
Randomization	All experiments were cell biological. Randomized assignment to groups was not relevant to our study. For microscopy, image fields containing cells were selected.
Blinding	Data collection and analysis was not blinded due to technical/personnel limitations. The nuclear channel was used identify fields containing cells to eliminate potential selection bias of specific markers. Image fields containing multiple cell structures were captured and all cell structures within fields were analyzed. Investigators were not blinded for analysis of experiments. Marker positions and intensities were analyzed using macros applied uniformly to all samples to exclude bias during analysis.

Reporting for specific materials, systems and methods

We require information from authors about some types of materials, experimental systems and methods used in many studies. Here, indicate whether each material, system or method listed is relevant to your study. If you are not sure if a list item applies to your research, read the appropriate section before selecting a response.

Materials & experimental systems

n/a	Involved in the study	
[ ]	[x]	Antibodies
[ ]	[x]	Eukaryotic cell lines
[x]	[ ]	Palaeontology and archaeology
[x]	[ ]	Animals and other organisms
[x]	[ ]	Human research participants
[x]	[ ]	Clinical data
[x]	[ ]	Dual use research of concern

Methods

n/a	Involved in the study	
[x]	[ ]	ChIP-seq
[x]	[ ]	Flow cytometry
[x]	[ ]	MRI-based neuroimaging

Antibodies

Antibodies used

Primary antibodies:
Par6B (Santa Cruz #sc-67393),
aPKC# BD Transduction #610175)
Par3 (Millipore #07-330)
CD13 (Abcam #ab108382)
E-cadherin (Cell Signaling #3195S)
ZO-1 (Cell Signaling #8193)
Ezrin (Cell Signaling #3145)
Pals1 (proteintech group #17710-1-AP)
beta-1-integrin (Abcam #ab30394)
V5 (Thermo Fisher Scientific # R960-25)
laminin (Abcam #ab11575)
alpha-Tubulin (Sigma #T9026)
Phalloidin (Invitrogen #A34055)
CD13 (Sigma # HPA004625)
Calreticulin (Abcam # ab92516)

GFP (Abcam, ab13970)

Secondary antibodies:
Alexa Fluor 488-Donkey anti-Rabbit, Jackson ImmunoResearch,711-545-152
Goat Anti-Mouse IgG Antibody (Cy3®), Jackson ImmunoResearch, 115-165-166
Alexa Fluor® 647-AffiniPure Donkey Anti-Mouse IgG (H+L), Jackson ImmunoResearch, 715-605-151

Validation

The Par6B (Santa Cruz #sc-67393) antibody has been validated by knockdown and genetic experiments (BenchSci). Western blot analysis of PARD6B expression in HeLa, Caki-1, MIA PaCa-2 , JAR, PC-3, and Jurkat whole cell lysates.

The aPKC# (BD Transduction #610175) antibody has been validated by overexpression experiments (BenchSci). From the vendor website: This antibody is routinely tested by western blot analysis. Other applications were tested at BD Biosciences Pharmingen during antibody development (from vendor website).

The Par3 (Millipore #07-330) antibody has been validated by overexpression and genetic experiments (BenchSci). From the vendor website: Evaluated by western blot on RIPA lysates from 3T3/A31, A431, and HeLa cells. Western Blot Analysis: 0.5-2 µg/mL of this antibody detected PAR-3 isoforms in RIPA lysates from 3T3/A31, A431, and HeLa cells.

Phalloidin (Invitrogen #A34055) has been validated in multiple cells lines (A549, BPAE, HCASM, HeLa, U2OS) from vendor website.

The aPKCi (BD Transduction #610175) antibody has been validated by overexpression experiments (BenchSci). This antibody is routinely tested by Rat Cerebrum Lysate in western blot analysis. Other applications were tested at BD Biosciences Pharmingen during antibody development (from vendor website). This antibody has also been validated by knockdown in our laboratory.

anti-CD13 (Abcam #ab108382) was validated in knockout THP-1 cells, (Immunohistochemistry, western blotting, immunoprecipitationon) from the vendor website and has been validated in Caco2 cells by knockdown experiments in our manuscript data (western blotting and immunofluorescence).

The CD13 (Sigma #HPA004625) antibody was validated in Caco2 cells by knockdown in our laboratory.

anti-Calreticulin (Abcam #ab92516) was validated in knockout HAP1 cells (Immunocytochemistry/ Immunofluorescence and westernblotting)(vendor website).

anti-ZO-1 (cell signaling #8193) antibody has been validated by relative expression experiments by western blotting and immunostaining (BenchSci). Species Reactivity in Human, Monkey(from vendor website). It was validated in Hep G2 cells by western blotting and immunoprecipitation.

anti-beta1 integrin (Abcam #ab30394) was validated in knockout HAP1 cells by immunostaining (from vendor website).

The E-cadherin (Cell Signaling #31955) antibody has been validated by relative expression overexpression and genetic experiments (BenchSci). From the vendor website: Western blot analysis of extracts from various cell lines using E-Cadherin (24E10) Rabbit mAb. Confocal immunofluorescent images of MCF7 cells using E-Cadherin (24E10) Rabbit mAb compared to an isotype control.
The Ezrin (Cell Signaling #3145) antibody has been validated by knockdown and genetic experiments (BenchSci). From the vendor website: Confocal immunofluorescent analysis of Hela cells labeled with Ezrin Antibody (green).

The Palsl (proteintech group #17710-1-AP) antibody has been tested in Y79 cells, human brain tissue by western blotting (vendor website). The expression of Pa Isl (proteintech group #17710-1-AP) by immunofluorescence were applied in Am J Physiol Cell Physiol. 2018 May 1; 314(5): C519-C533.

The beta-1-integrin (Abeam #ab30394) antibody has been validated by genetic experiments (BenchSci). This monoclonal antibody to integrin beta 1 has been knockout validated in ICC/IF and flow cytometry. The expected signal was observed in wild type cells and was not seen in knockout cells. (vendor website).

The VS (Thermo Fisher Scientific# R960-25) antibody has been validated by overexpression experiments (BenchSci). From the vendor website: This antibody is functionally tested against 20 ng of an E. coli expressed fusion protein containing a VS epitope using a chemiluminescent substrate at a 1-minute exposure. This antibody has also been tested in Western blot against 25 ng of recombinant Positope'M protein. The Positope'M control protein is a 53 kDa recombinant protein that contains seven epitope tags, including His (C-term), HisG, c-myc, and VS. Low background was observed using chemiluminescent or alkaline phosphatase reagents for detection. Using chemiluminescence as the detection method, no cross-reactivity has been observed in bacterial lysates. In mammalian lysates, a few cross-reactive proteins have been observed upon overexposure of blots. This antibody has also been used successfully to immunoprecipitate fusion proteins that contain the VS epitope. VS (Thermo Fisher Scientific# R960-25) antibody has been used for ELISA, ICC, IF, IP, WB.

The laminin (Abeam #ab11575) antibody has been validated by genetic experiments (BenchSci). From the vendor website: lmmunofluorescence showing co-localization of CL-Ll and Laminin in E13.5 mouse embryos sections. CL-Ll is expressed in the basal membrane of the epithelium in the palate shelf of the maxilla. In contrast La min in expression is present all around the epithelium membrane.

The a-Tubulin (Sigma #T9026) antibody has been enhanced validated by the vendor and is widely used for western blotting and immunofluorescence. From the vendor website: Enhanced Validation-By Independent Antibodies Chicken fibroblasts cells were fixed and permeabilized with methanol followed by acetone. Fixed cells were stained with 1:1,000 Monoclonal Anti-a-Tubulin antibody produced in Mouse, Clone: DM1A (Cat. No. T9026) or 1 µg/ml Monoclonal Anti-a-Tubulin antibody produced in Mouse, Clone: B-5-1-2 (Cat. No. T5168). Two Anti-a-Tubulin antibodies, T9026, and T5168 target different regions of a-Tubulin show similar staining profiles between the two antibodies, demonstrating Independent Antibody Verification.

The expression of flag 1/1000 (Delta Bio labs# DB125) by western blotting were applied in the paper- Ricotta D, Hansen J, Preiss C, Teichert D, Honing S. 2008. Characterization of a protein phosphatase 2A holoenzyme that dephosphorylates the clathrin adaptors AP-1 and AP-2. J Biol Chem. Feb 29;283(9):5510-7.

The myc 1/1000 (Origene # TA150121) antibody was validated by several experimental data from the vendor website (doi: 10.1093/bra in/awv00S ;doi: 10.1371/journal .pone.01163 72;
doi:10.1074/jbc.M113.546077;) and was applied in WB, flow cytometry, IP, IF.

The GFP 1/1000 (Abeam #ab13970) antibody was validated by several experimental data from the vendor website (doi: 10.1371/journal.pone.0095236; DOI 10.1074/jbc.M112.350884) and was applied in IHC, WB, IF.
anti-V5 (Thermo Fisher Scientific # R960-25) was validated by overexpression in HEK293 and Caco2 cells for western blotting and immunostaining in our laboratory.

Eukaryotic cell lines

Policy information about cell lines

Cell line source(s)	Caco2 and HEK293T cells were from ATCC.
Authentication	Caco2 cells were authenticated by STR analysis. HEK293T cells were not authenticated.
Mycoplasma contamination	All cell lines were negative for mycoplasma.
Commonly misidentified lines (See ICLAC register)	None.
